# Plaster of Paris in the Treatment of Fractures

**Published:** 1885-07

**Authors:** W. H. Elliot

**Affiliations:** Chairman, Savannah, Ga.


### PLASTER OF PARIS IN THE TREATMENT OF FRACTURES.

REPORT ON SURGERY FROM THE FIRST CONGRESSIONAL DISTRICT TO THE MEDICAL ASSOCIATION OF GEORGIA, AT ITS THIRTY-SIXTH ANNUAL SESSION AT SAVANNAH, APRIL, 1885, BY W. H. ELLIOT, M. D., CHAIRMAN, SAVANNAH, GA.

That I can find in no standard work on surgery any satisfactory exposition of the best method of using plaster of Paris in the treatment of fractures must be my excuse for bringing this subject before the Association. As usually presented, plaster of Paris is simply classed as one of the forms of immovable dressings, generally commended for its ready applicability and accurate fit. So long as this estimate is applied to the plaster *bandage* only, I take no exception. Plaster, used in the form of a bandage, is undoubtedly one of the best, if not *the* best, form of immovable apparatus, and possesses all the disadvantages of that method of treatment. Chief among these is, that the limb is entirely encircled by a firm, unyielding substance, which will allow no swelling, is liable to cause gangrene, and therefore ought not to be applied till inflammation begins to subside.

I must here record my protest against the practice of putting a bandage around a broken limb before inflammation has taken place, except as a temporary expedient, for the removal of a patient, or support of a limb, till other materials can be obtained. If, then, it is unsafe to apply the ordinary forms of immovable apparatus to recent fractures, we are unable to obtain immobility, just when that is essential for controlling inflammation, lessening pain and securing union.

Now, there is another method of using plaster of Paris, which I claim is not open to these objections, being applicable to recent fractures without risk of interfering with the circulation. I refer to the splint first brought to the notice of the profession by Dr. James L. Little, of New York, many years ago. The plaster of Paris splint holds a position, intermediate between the ordinary splints, which are usually applied to recent fractures, and the various forms of immovable apparatus used later on. It differs from the ordinary splint, in that it can retain itself and the injured limb in position, without the use of a bandage during the critical period of inflammation, when, as I have already stated, the application of a bandage is unsafe. On the other hand, it differs from the other forms of immovable apparatus in securing complete immobility, without the risk of interfering with the circulation.

This splint is made with canton flannel, made rigid by plaster and accurately fitted to the injured limb, encircling not more than four-fifths of its circumference, thus leaving, on any aspect of the limb selected by the operator, an uncovered strip as long as the splint itself.

In making the splint the first requisite is good plaster. This can be had by taking from the centre of a fresh barrel.

First, fit a piece of canton flannel from the extremity of the broken limb to the first joint above the seat of fracture, but only partially around it. Lay this on a table and cut out one to three more thicknesses of flannel, according to the size of the limb. Cut out two thicknesses of cotton wadding an inch larger all around than the pieces of flannel. Mix the plaster, freed of all lumps, with a little less than its volume of cold water. Stir the pieces of cloth successively in the mixture till they are thoroughly wet with it. Spread the cloth, piece after piece, on a table, smoothing the plaster on each one before the next is laid on it. On top of the last piece lay the cotton wadding; apply the splint at once in its soft condition to the broken limb, holding the edges of the splint as close together as possible, to secure perfect coaptation without wrinkles, while the splint is secured with a bandage which should be firmly applied. Then set the fracture, making the proper manipulation of the limb, including extension and counter-extension, which must be maintained till the plaster is set. This will usually take about fifteen minutes. Then take off the bandage. The injured limb is now lying in an immovable, well-cushioned splint, which, if properly applied, can be left on till union is complete. The splint being left open its whole length, no constriction of the limb can take place, and the surgeon can at any time inform himself as to the state of the circulation and general condition of the injured parts. As soon as the swelling begins to subside, the splint, which is somewhat elastic, can be drawn in by successive applications of a bandage. A little starch added will prevent this from slipping and is a very convenient mode of bracing the splint, if the ability of the patient to get about renders this desirable. If the cotton wadding wears thin, the splint can generally be taken off without sacrificing its integrity, and, after being newly lined, re-applied.

The advantages of this splint, summed up, are: safe application to recent fractures without risk of gangrene; perfect adaptability; a splint can be made for any surface to which a piece of cloth can be fitted; great comfort to the patient; small liability to produce excoriations; after being once well fitted it is not apt to get out of position, and so requires less work from the surgeon than any other form of apparatus; general applicability; being adapted to all fractures except those of the skull, clavicle ribs and forearm. Lastly, I think it attains the best results. I cannot establish this point by the production of statistics, but I have frequently, by the use of the plaster splint, obtained union without shortening in fractures of the femur, a result I have obtained by no other apparatus.

It only remains to be said that my conclusions are based upon twelve years' experience.

